# Brain region‐specific neuromedin U signalling regulates alcohol‐related behaviours and food intake in rodents

**DOI:** 10.1111/adb.12764

**Published:** 2019-05-08

**Authors:** Daniel Vallöf, Aimilia Lydia Kalafateli, Elisabet Jerlhag

**Affiliations:** ^1^ Institute of Neuroscience and Physiology, Department of Pharmacology The Sahlgrenska Academy at the University of Gothenburg Gothenburg Sweden

**Keywords:** addiction, dopamine, gut‐brain axis, reinforcement, reward

## Abstract

Albeit neuromedin U (NMU) attenuates alcohol‐mediated behaviours, its mechanisms of action are poorly defined. Providing that the behavioural effects of alcohol are processed within the nucleus accumbens (NAc) shell, anterior ventral tegmental area (aVTA), and laterodorsal tegmental area (LDTg), we assessed the involvement of NMU signalling in the aforementioned areas on alcohol‐mediated behaviours in rodents. We further examined the expression of *NMU* and NMU receptor 2 (*NMUR2*) in NAc and the dorsal striatum of high compared with low alcohol‐consuming rats, as this area is of importance in the maintenance of alcohol use disorder (AUD). Finally, we investigated the involvement of NAc shell, aVTA and LDTg in the consumption of chow and palatable peanut butter, to expand the link between NMU and reward‐related behaviours. We demonstrated here, that NMU into the NAc shell, but not aVTA or LDTg, blocked the ability of acute alcohol to cause locomotor stimulation and to induce memory retrieval of alcohol reward, as well as reduced peanut butter in mice. In addition, NMU into NAc shell decreased alcohol intake in rats. On a molecular level, we found increased *NMU* and decreased *NMUR2* expression in the dorsal striatum in high compared with low alcohol‐consuming rats. Both aVTA and LDTg, rather than NAc shell, were identified as novel sites of action for NMU's anorexigenic properties in mice based on NMU's ability to selectively reduce chow intake when injected to these areas. Collectively, these data indicate that NMU signalling in different brain areas selectively modulates different behaviours.

## Graphical abstract

NMU signalling in different brain areas selectively modulates different behaviours. Indeed, NMU into the NAc shell, but not aVTA or LDTg, reduced alcohol intake in rats as well as blocked the ability of acute alcohol to cause locomotor stimulation and to induce memory retrieval of alcohol reward, and reduced peanut butter in mice. We found increased NMU and decreased *NMUR2* expression in the dorsal striatum in high compared to low alcohol‐consuming rats. NMU into aVTA or LDTG reduce chow intake.

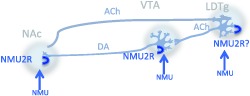

## 1 INTRODUCTION

Neuromedin U (NMU) is a highly conserved neuropeptide with pleiotropic functions. It is well‐known for its ability to reduce food intake and decrease the motivation to consume foods (for review^1^). NMU receptors 2 (NMUR2) located in the arcuate nucleus, paraventricular nucleus, and dorsal raphe nucleus are key players for NMU's anorexigenic properties.^2^, ^3^, ^4^, ^5^, ^6^ An additional study has established that NMU infusion into the third ventricle attenuates both the acute effects of alcohol in mice and alcohol intake in a chronic rat model of alcohol use.^7^ The role of brain region specific NMUR2 in the acute and chronic effects of alcohol has not yet been explored.

Alcohol‐mediated behaviours are mandated via alcohol's ability to activate the nucleus accumbens (NAc) shell (for review^8^). In addition, this area contains NMU,^9^ the expression of *NMUR2* has been identified on GABA terminals in NAc,^4^ and the NMUR2 protein has been detected within NAc.^10^ We therefore hypothesise that activation of NMUR2 in NAc shell attenuates acute, as well as chronic effects of alcohol in rodents. Initial experiments explored the effects of NMU infusion into the NAc shell, on the ability of acute alcohol to cause locomotor simulation and to induce reward‐dependent memory retrieval in the conditioned place preference (CPP) model in mice, which are known to robustly respond to alcohol in these models.^7^ To further investigate the involvement of NAc shell as a mediator of the acute NMU‐alcohol link, we investigated changes in *cFos* expression, an immediate‐early gene and indicator of neuronal activity,^11^ following acute alcohol injection after central pretreatment of vehicle or NMU in rats. Moreover, the ability of NMU‐NAc shell to reduce alcohol intake in rats consuming physiological relevant alcohol levels^12^ for 12 weeks was evaluated. Evidence that the behavioural change from recreational rewarding alcohol use to compulsive and habitual use involves a neuronal shift from NAc shell to the dorsal striatum,^13^ led us to investigate expression of *NMU* and *NMUR2* in NAc as well as dorsal striatum in high compared with low alcohol‐consuming rats.

Besides striatum, the acute rewarding effects of alcohol involve activation of the cholinergic projections from laterodorsal tegmental area (LDTg) to the ventral tegmental area (VTA).^14^, ^15^ The VTA is a heterogeneous area, including the anterior (aVTA) as well as posterior part of the VTA. When it comes to the aVTA, studies have found that alcohol infusion into this part increases accumbal dopamine in rats^16^ and that nicotinic acetylcholine receptors herein regulate alcohol‐mediated behaviour in rodents.^17^, ^18^, ^19^ Recently, NMUR2 was detected in the VTA^10^; however, neither *NMU* nor *NMUR2* expression has been investigated in LDTg. Both aVTA and LDTg were identified as important areas for the gut‐brain peptide ghrelin to modulate alcohol drinking in mice.^20^ Given the link between alcohol‐related behaviours and the aVTA and LDTg, the possibility should be considered that NMU signalling within the aforementioned areas modulates alcohol‐induced activation of the mesolimbic dopamine system. We therefore evaluated the effects of NMU infusion into the aVTA or LDTg on acute alcohol‐induced locomotor stimulation, as well as on memory of alcohol reward in the CPP model in mice.

Additionally, brain region specific NMUR2 regulate the pleotropic functions, including food intake and alcohol‐related behaviours.^7^, ^21^, ^22^ Therefore, food preference studies in mice were conducted, where chow and peanut butter intake was investigated following NMU infusion into NAc shell, aVTA, or LDTg. Collectively, the present study contributes to further understanding of the involvement of NMU in reward processes, with focus on striatal signalling.

## 2 MATERIAL AND METHODS

### 2.1 Experimental procedure

Previous studies have established that central NMU infusion blocks the acute behavioural responses of alcohol, as measured by locomotor stimulation, accumbal dopamine release, and CPP in mice and reduces alcohol intake in rats consuming alcohol for longer periods of time.^7^ In line with this, the present experiments were designed to evaluate the functional role of brain region specific NMUR2 that are crucial for acute and chronic alcohol‐mediated behaviours in mice and rats, respectively. Moreover, mice as well as rats display a robust effect of alcohol in the models used herein.^7^, ^20^

### 2.2 Animals

For the acute effects of alcohol (locomotor activity and CPP), the tests were conducted in male NMRI mice, as they display a robust alcohol‐induced locomotor stimulation, alcohol CPP,^20^ and peanut butter intake.^23^ They were maintained on a 12/12‐hour light/dark cycle (20°C and 50% humidity). The mice were tested during their light phase, as the ability of alcohol to evoke behaviour is robustly obtained during the light (mice's resting phase). For the chronic studies with alcohol (the intermittent access paradigm), male outbred Rcc Han Wistar rats known to voluntary consume alcohol causing pharmacological relevant blood alcohol concentrations^12^ were used. The rats were kept on a 12‐hour reversed light dark cycle (lights off at 8 AM) in rooms with a 20°C and 50% humidity. In these rats, the drug was administered prior to the onset of the dark cycle and before bottles' presentation, as rats consume the highest levels of alcohol during this initial dark phase. Different phases of the sleep/wake cycle are thus necessary for obtaining robust alcohol responses. Mice and rats respond similarly to various gut‐brain peptides including ghrelin, NMU, glucagon‐like peptide‐1, and amylin in regards to alcohol‐mediated behaviours measured in the light as well as dark phase (for review, see Jerlhag^24^). Mice and rats were used in the present study, based on studies reporting a robust effect of alcohol in the animal models used herein, with a similar response to NMU treatment.^7^

### 2.3 Drugs

Alcohol (96%; VWR International AB, Stockholm, Sweden) was diluted in vehicle (0.9% NaCl) and was administered at a dose of 1.75 g/kg intraperionetally (ip) 5 minutes prior to initiation of the experiments, as this displays a robust activation of the mesolimbic dopamine system.^20^ NMU was diluted in vehicle (Ringer solution; NaCl 140 mM, CaCl_2_ 1.2 mM, KCl 3.0 mM, and MgCl_2_ 1.0 mM; Merck KGaA, Darmstadt, Germany). NMU was administrated locally and bilaterally into the specific reward area (NAc, aVTA, or LDTg) 20 minutes prior to alcohol exposure, as this was the time frame necessary for central NMU to attenuate alcohol's and amphetamine's behavioural effects in rodents.^7^, ^25^ The selected dose of 62.5 ng into the NAc shell has no effect per se but is able to prevent amphetamine‐induced locomotor stimulation.^25^ In rats, a higher dose of 250 ng per side into NAc shell was used since this dose had no effect on gross behaviour in a pilot experiment (Supplementary Figure 1A). This NMU dose is three times lower (0.3 nmol) than the one used in previous rat studies.^4^, ^5^ In mice, pilot studies revealed that an NMU dose of 125 ng per side into the aVTA (Supplementary Figure 1B) and 250 ng per side into LDTg (Supplementary Figure 1C) had no effect per se on either visually assessed gross behaviour or locomotor activity. In addition, these pilot experiments reveal that higher doses of NMU affect locomotor activity and visual observation of gross behaviour per se, so the outcome of further studies with higher doses is difficult to interoperate. Different doses were used for NAc shell, aVTA, and LDTg because of tentatively different NMU sensitivity in these areas. For all local and bilateral infusions, a volume of 0.5 μl per side was administered over 1 minute. The injector was left in place for another minute and was then retracted.

### 2.4 Guide implantation

Identical procedure, coordinates, and verification of placements as previously described^26^, ^27^ (Supplementary material 1; Supplementary figure 2A‐D).

### 2.5 Acute effects of alcohol in mice

#### 2.5.1 Locomotor activity experiments

Locomotor activity was registered in eight‐sound attenuated, ventilated, and dim lit locomotor boxes (420 × 420 × 200 mm, Kungsbacka mät‐ och reglerteknik AB, Fjärås, Sweden). Photocell beams at the floor level allowed a computer‐based system to register accumulated number of new photocell beams interrupted during a 60‐minute period. In each experiment, the mice were allowed to habituate to the locomotor activity box 1 hour prior to drug challenge.

Initially, the effect of bilateral NMU (62.5 ng per side) administration into the NAc shell, on alcohol‐induced (1.75 g/kg, ip) locomotor stimulation was evaluated. Thereafter, the effect of NMU either into the (a) aVTA (125 ng per side) or (b) LDTg (250 ng per side) on alcohol‐induced (1.75 g/kg, ip) locomotor stimulation was studied. For each brain area, the following treatment groups were created: Veh‐Veh, Veh‐Alc, NMU‐Veh, or NMU‐Alc.

#### 2.5.2 Conditioned place preference

As described previously,^7^ a two‐chambered CPP apparatus (45 lux) with distinct visual and tactile cues was used. Each CPP test consisted of preconditioning (day 1), conditioning (days 2 to 5), and post‐conditioning (day 6). The initial place preference was determined after the mice were placed in the middle of the CPP box with 20‐minute free access to both compartments during preconditioning. Conditioning (20 min per session) was done using a biased procedure in which alcohol (1.75 g/kg, ip) was paired with the least preferred compartment and vehicle with the preferred compartment. All mice received one alcohol and one vehicle injection everyday, and the injections were altered between morning and afternoon in a balanced design. At the post‐conditioning day, the mice were either injected with NMU or an equal volume of vehicle locally and bilaterally into either the (a) NAc shell (62.5 ng per side), (b) aVTA (125 ng per side), or (c) LDTg (250 ng per side). They were then placed on the midline between the two compartments with free access to both compartments for 20 minutes (creating the following treatment groups for each of the three experiments; Alc‐Veh and Alc‐NMU). The present design of the CPP paradigm evaluates reward‐dependent memory retrieval of alcohol reward as NMU was infused at the post‐conditioning day. CPP was calculated as the difference in percentage of total time spent in the drug‐paired (ie, less preferred) compartment during the post‐conditioning and the preconditioning session. In addition, three separate control experiments were conducted to evaluate the effect of NMU in the NAc shell, aVTA, or LDTg on CPP per se. These mice were subjected to the same procedure but received vehicle injections instead of alcohol throughout the conditioning (nonalcohol conditioned). At post‐conditioning, the mice were injected locally and bilaterally with NMU or an equal volume of vehicle into the NAc shell, aVTA, or LDTg. The mice were then placed on the midline between the two compartments with free access to both compartments for 20 minutes (creating the following two treatment groups Veh‐Veh and Veh‐NMU for each of the three experiments).

Although it would be interesting to evaluate the effect of NMU into either area on alcohol reward as well as conditioned taste aversion, these studies cannot be conducted because of ethical limitations and animal discomfort. In such tests, NMU would precede alcohol/vehicle injections on each conditioning day, altering baseline behaviour of the rodents, which are influenced negatively following two local infusions on a daily basis.

### 2.6 Chronic effects of alcohol in rats

#### 2.6.1 Intermittent access 20% alcohol two‐bottle‐choice drinking paradigm

Two separate alcohol intake experiments were conducted to evaluate the (a) effect of NMU on alcohol intake in high alcohol‐consuming rats and (b) expression levels of *NMU* and *NMUR2* in high versus low alcohol‐consuming rats. The cut‐off for low versus high alcohol‐drinking rats was 3.5 g/kg/day. In both experiments, rats had free access to one bottle of 20% alcohol and one bottle of water during three 24‐hour sessions per week for 12 weeks prior to the initiation of the experiments (Mondays, Wednesdays, and Fridays).^12^ Alcohol, water, and food intake, as well as preference for alcohol were registered at 24 hours after bottle presentation. The days in between the rats had unlimited access to two bottles of water.

#### 2.6.2 Experiment 1—effects of NMU into NAc shell on alcohol intake in rats

The findings that NMU into the NAc shell prevents alcohol‐induced locomotor stimulation and CPP, led us to investigate the effects of NMU (250 ng per side) or vehicle infusion into the NAc shell on alcohol intake in high alcohol‐consuming rats. Only high alcohol‐consuming rats were included here as a previous study shows that high‐ but not low‐alcohol consuming rats display a reduction in alcohol intake after NMU administration.^7^ Only 24 hours intake was measured as our previous study displayed a robust reduction in the 24‐hour alcohol intake following central NMU administration.^7^ In our hands, previous studies have shown that ghrelin receptor antagonists, glucagon‐like peptide‐1 receptor agonists, NMU, as well as amylin receptor agonists block alcohol‐induced locomotor stimulation and CPP, also reducing alcohol intake in rats (for review, see Jerlhag^24^). To minimize the number of rats used, the effects of NMU into aVTA or LDTg on alcohol intake were not investigated, as our mice studies do not reveal that NMU signalling in these areas modulates alcohol‐induced locomotor stimulation or CPP.

#### 2.6.3 Experiment 2—NMU and NMUR2 expression in low and high alcohol‐consuming rats

To further link NMU signalling in the striatum with alcohol‐related behaviours, the expression of *NMU* and *NMUR2* in the NAc and dorsal striatum were explored. Following 12 weeks of intermittent access to alcohol, rats were decapitated, brains were rapidly removed and immediately placed on a cold glass plate. NAc and dorsal striatum were rapidly dissected, transferred into a plastic tube, snap froze, and stored in −80°C until further analysis. In addition, as a control, the expression of *NMU* and *NMUR2* in high versus low alcohol‐consuming rats was evaluated in other areas important for addiction processes, namely, VTA, amygdala, hippocampus, and prefrontal cortex.^28^ When obtaining all of these areas, dissection rather than isolation of brain punches has to be used. Because of technical limitations with the method followed here, the whole NAc was obtained with no further distinction of NAc core and shell. RNA extraction and qPCR were performed as described in Supplementary material 2. For the analysis, the selected reference genes (RG) were *HMBS* and *YWHAZ*, and the genes of interest (GOI) were *NMU* and *NMUR2*. For all statistical analyses, the ΔC_T_ values were used. The ΔΔC_T_ values were calculated as the average ΔC_T_ of the internal calibrator (low alcohol group) subtracted from the average ΔC_T_ of the experimental group (high alcohol group). For better visual representation and comprehension, all expression data graphs demonstrate fold change expression levels (2^‐ΔΔCt^). In this experiment, the internal calibrator was the low alcohol‐consuming group compared with the experimental group of the high alcohol consumers.

### 2.7 Acute effects of alcohol in rats

#### 2.7.1 cFos expression in nucleus accumbens shell

Alcohol and NMU have independently been shown to activate neurons of NAc shell, as measured by *cFos*, in rats.^4^ However, the interaction between NMU and alcohol on neuronal activity in NAc shell is unknown. Rat experiments were conducted to evaluate this, by measuring the expression of the neuronal activation marker *cFos*. In addition to NAc shell, *cFos* expression was evaluated in NAc core as a control test, as this area does not respond to acute alcohol with dopamine release.^29^, ^30^ Rats were treated acutely with NMU (1 μg) or vehicle into the third ventricle prior to alcohol exposure (1.75 g/kg, ip) creating the following two treatment groups: Veh‐alcohol and NMU‐alcohol. Since the focus of the present study was to investigate the interaction between NMU and alcohol, vehicle‐vehicle and NMU‐vehicle rats were not included, in order to reduce the number of animals used. Twenty minutes following alcohol injections, where the behavioural response to alcohol is profound, rats were decapitated, and the brains were removed and snap frozen in plastic tubes in −80°C until further analysis. In the present project, the interaction between NMU and alcohol in the NAc shell was investigated, and therefore, brain punching isolation was used. For the isolation of NAc shell and core regions, brains were placed in a cold rat brain matrix (Zivic instruments, Pittsburg, PA, USA) and coronally sectioned in 1 mm slices rostral to the fusion of the optic nerves with the optic chiasm according to the brain atlas.^31^ The desired section was placed under a stereoscope on a very cold glass plate (mix of dry ice and regular ice) to avoid tissue degradation, and core and shell accumbal tissue was isolated from both sides, using a tissue biopsy punch (Zivic instruments, Pittsburg, PA, USA). Rats were used instead of mice in the present experiment, as it is technically easier to extract both NAc shell and core. RNA preparation and PCR were performed as described in Supplementary material 2. The corrected C_T_ values raw data were analysed using the comparative C_T_ method as previously described.^32^ In these experiments, the selected RG were *HMBS* and *YWHAZ*, and the GOI was c*Fos*. In brief, the individual ΔC_T_ values were calculated as C_Τ *GOI*_ − C_T RG_. Statistical analysis and data representation are same as above (see experiment 2*—*NMU and NMUR2 expression in low and high alcohol‐consuming rats). As the present experiment aimed at investigating the link between NMU and alcohol in the NAc shell further, the vehicle‐alcohol group was used as an internal calibrator. Only vehicle‐alcohol and NMU‐alcohol rats were included to minimize the number of rats used.

### 2.8 Food consumption studies in mice

Food consumption studies in mice^33^ were conducted to evaluate the possibility that different subpopulations of NMUR2, specifically, those in NAc shell, aVTA, and LDTg; modulate food intake; and alcohol‐mediated behaviours. As described previously, the group‐housed mice had unlimited access to chow and peanut butter for 3 days prior to and 4 days after surgery (Crunchy, Green Choice).^23^ This allows the mice to acclimatize to the taste of peanut butter. At the test day, the mice were allowed to habituate to a sterile cage for 1 hour. Thereafter, NMU or an equal volume of vehicle was infused locally and bilaterally into either the (a) NAc shell (62.5 ng per side), (b) aVTA (125 ng per side), or (b) LDTg (250 ng per side). Preweighed chow and peanut butter were thereafter placed inside the cage, and the food was reweighed 4 hours later since previous data show a robust food consumption at this time point.^23^

### 2.9 Statistical analysis

The locomotor activity experiments were evaluated by a one‐way ANOVA followed by Bonferroni post‐hoc test for comparisons between different treatments, and the CPP and food intake experiments were evaluated by an unpaired *t* test. An unpaired *t* test was performed on the ΔC_T_ values for the investigation of *NMU*, *NMUR2*, and c*Fos* expression, and the Pearson correlation test was performed to analyse the correlation between *NMU*/*NMUR2* expression patterns and the mean values of alcohol intake in that group of rats.

## 3 RESULTS

### 3.1 Effect of intra‐NAc‐NMU on alcohol‐induced locomotor stimulation, memory retrieval of alcohol reward in the CPP paradigm, and food intake in mice

**Figure 1:**
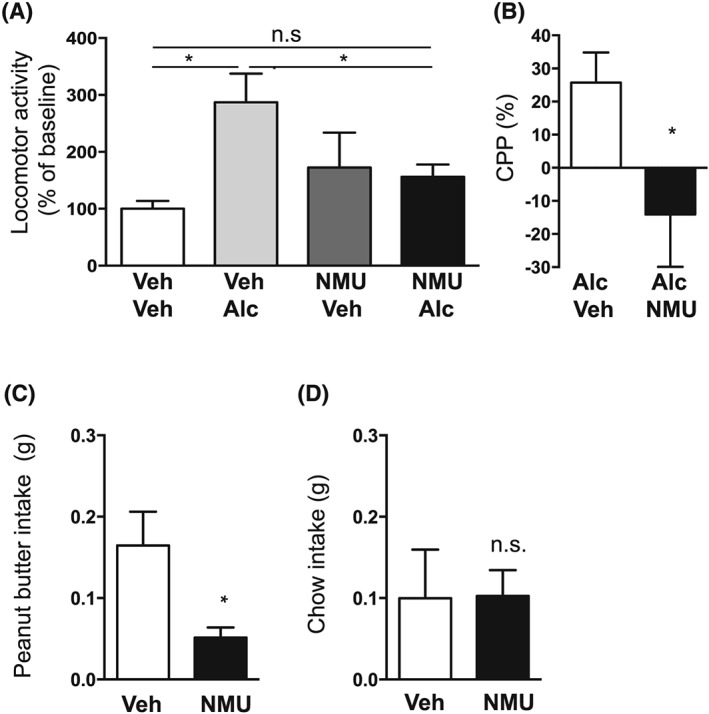
Effects of neuromedin U (NMU) infusion into nucleus accumbens (NAc) shell on alcohol‐induced locomotor stimulation, conditioned place preference (CPP), and food intake in mice. A, The alcohol (Alc)‐induced (1.75 g/kg, ip) locomotor stimulation was attenuated by NMU (62.5 ng per side) into NAc shell compared with vehicle (Veh), at a dose with no effect per se. B, Compared with vehicle, intra‐NAc infusion of NMU inhibited the alcohol‐induced CPP. C, NMU into NAc shell reduced peanut butter intake compared with vehicle. D, Chow intake was not altered by NMU into NAc shell. (Data are presented as mean ± SEM, *P < .05, n.s. *P* > .05).

An overall main effect of treatment was found on locomotor activity in mice following systemic administration of alcohol and intra‐NAc injection of NMU (*F*_[3,28]_ = 3.79, *P* = .0212). As shown in Figure 1A, post‐hoc analysis revealed that alcohol (n = 10) increased locomotor activity compared with vehicle (*P* < .05, n = 8). The alcohol response was lower in NMU (n = 7) treated mice compared with vehicle treated mice (*P* < .05), while there was no difference in locomotor activity response in vehicle treated mice and NMU‐alcohol treated mice (*P* > .05), showing that accumbal NMU attenuates alcohol‐induced locomotor stimulation. The selected dose of NMU had no effect per se on locomotor activity compared with vehicle treatment (*P* > .05, n = 7). Due to misplacement, one mouse was excluded in the NMU‐vehicle as well as in the NMU‐alcohol groups.

The alcohol‐induced reward memory retrieval in the CPP test (n = 7) was significantly attenuated by NMU into the NAc shell (n = 5) on the post‐conditioning day (*P* = .0287, Figure 1B). Control experiments showed that intra‐NAc NMU (14 ± 14%) administration had no effect per se on the CPP compared with vehicle treatment (5 ± 13%, *P* = .6639, n = 7 per group). Due to misplacement, one mouse was excluded in the vehicle‐vehicle, vehicle‐NMU, alcohol‐NMU groups, and two mice were excluded in the NMU‐alcohol group.

Intra‐NAc NMU administration reduced peanut butter intake compared with vehicle treatment (*P* = .0.0143, n = 15 per group, Figure 1C). On the contrary intra‐NAc, NMU had no effect on chow intake (*P* = .9687, Figure 1D). Due to misplacement, one mouse was excluded in each group.

### 3.2 Effect of intra‐NAc NMU on alcohol intake in high alcohol consuming rats

**Figure 2:**
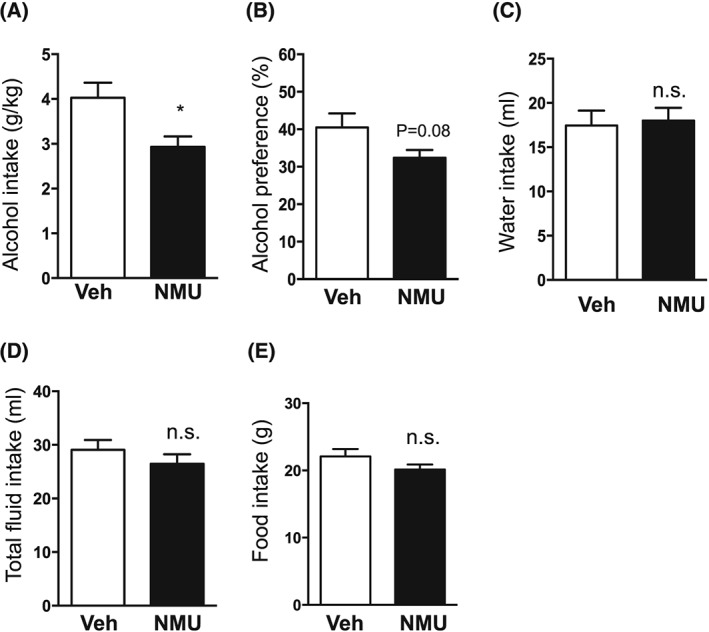
Neuromedin U (NMU) infusion into nucleus accumbens (NAc) shell and alcohol intake in high alcohol‐consuming rats. A, Compared with vehicle (Veh) treatment, NMU infusion into NAc shell (250 ng per side) reduced alcohol intake in rats consuming high amounts of alcohol for 12 weeks. B, There was a tendency in reduced preference for alcohol over water by NMU into NAc shell. NAc‐NMU did not affect C, water intake, D, total fluid intake, or E, food intake. (Data are presented as mean ± SEM, *P < .05, n.s. *P* > .05.).

There was no difference (*P* = .4891) in the 12‐week baseline alcohol consumption in rats later subjected to vehicle (3.8 ± 0.2 g/kg, n = 7) or NMU (4.1 ± 0.4 g/kg, n = 7) treatment. NMU into NAc shell reduced alcohol intake (Figure 2A, *P* = .0201) but not alcohol preference (Figure 2B, *P* = .0812), compared with vehicle. On the contrary, intra‐NAc NMU administration had no effect on water intake (Figure 2C, *P* = .8067), total fluid intake (Figure 2D, *P* = .3234), or food intake (Figure 2E, *P* = .1672).

### 3.3 Effect of long‐term alcohol exposure on expression of NMU and NMUR2 in rats

**Figure 3:**
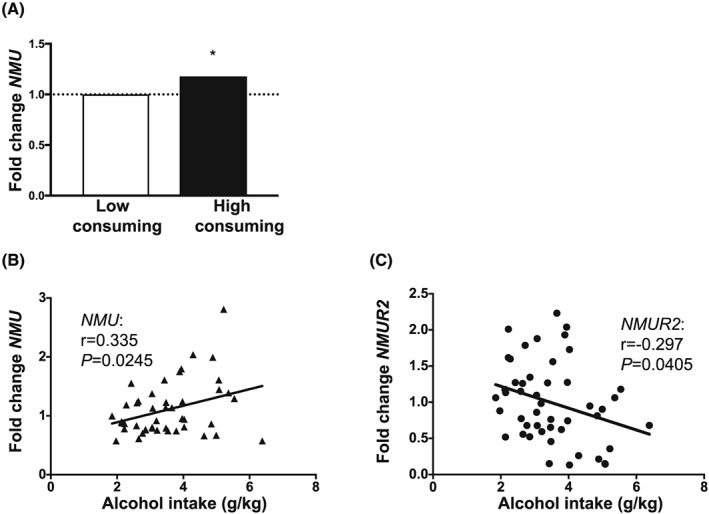
Expression levels of neuromedin U (*NMU*) and NMU receptor 2 (*NMUR2*) in dorsal striatum in high versus low alcohol‐consuming and *cFos* expression in nucleus accumbens (NAc) shell after acute injection of NMU and alcohol in rats. A, Expression of *NMU* in dorsal striatum is increased in high compared with low alcohol‐consuming rats. B, Positive correlation between *NMU* expression in dorsal striatum and mean values of alcohol intake and C, negative correlation between *NMUR2* expression in dorsal striatum and alcohol intake. (*P < .05 for the expression and correlation. Data are presented as fold change in the form of 2^‐ΔΔCT^).

To evaluate the effects of long‐term alcohol consumption on the expression of *NMU* and *NMUR2*, rats that had voluntarily consumed alcohol for 12 weeks were divided in low and high consumers based on their level of alcohol consumption (cut‐off >3.5 g/kg). In the dorsal striatum, a significant effect of alcohol consumption was noted, with higher *NMU* expression in the high compared with low consuming rats (low consumers n = 23; high consumers n = 22; *t*(43) = 2.7, *P* = .0099, Figure 3A).

Separate analysis revealed a significant positive correlation between *NMU* expression in the dorsal striatum and alcohol intake (*r* = .335; n = 45; *P*  =  .0245, Figure 3B). Also, a significant negative correlation was found between *NMUR2* expression, in the dorsal striatum, and alcohol intake (*r* = −.297; n =  48; *P* = .0405, Figure 3C).

There were no differences in *NMU* expression in the NAc, VTA, PFC, amygdala, or hippocampus (Supplementary table 1), or on *NMUR2* expression in the NAc, VTA, PFC, amygdala, hippocampus, or dorsal striatum (Supplementary table 2) in high compared with low alcohol‐consuming rats.

### 3.4 cFos expression in NAc shell and core after acute injection of alcohol and NMU in rats

To evaluate the effects of neuronal activation as measured by *cFos* expression in the NAc shell, rats received an acute alcohol injection after pretreatment with vehicle (n = 7) or NMU (n = 7). In the NAc shell, a significant effect was noted, with higher *cFos* expression in rats pretreated with NMU compared with vehicle pretreated (*t*[12] = 2.1, *P* = .0272, Supplementary Figure 3). As hypothesised, there were no differences in *cFos* expression in the NAc core, between the two treatment groups (*t*[12]  =  1.6, *P*  =  .0704, vehicle‐alcohol 0.68 ± 0.19, NMU‐alcohol 0.27 ± 0.17). Because of technical errors, one mouse was excluded in each group.

### 3.5 Effects of intra‐aVTA administration of NMU on alcohol‐induced locomotor stimulation, memory retrieval of alcohol reward in the CPP paradigm as well as food intake in mice

**Figure 4:**
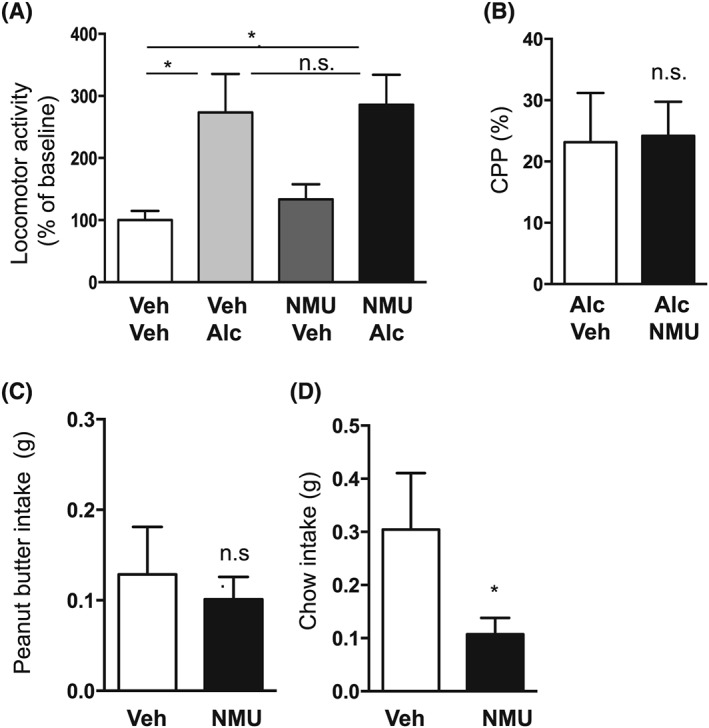
Effects of neuromedin U (NMU) infusion into the anterior ventral tegmental area (aVTA) on alcohol‐related behaviours and food intake in mice. A, Alcohol (Alc)‐induced (1.75 g/kg, ip) a locomotor stimulation in mice pretreated with vehicle (Veh) as well as NMU (125 ng per side) into the aVTA. There was no difference in alcohol response in the Veh‐Alc and NMU‐Alc groups. The selected NMU dose had no effect per se on locomotor activity. B, There were no differences in alcohol‐induced conditioned place preference (CPP) response in intra‐aVTA vehicle (Alc‐Veh) and NMU (NMU‐Alc) treated mice. C, Compared with vehicle, infusion of NMU into aVTA did not affect peanut butter intake but D, decreased chow intake in mice. (Data are presented as mean ± SEM, *P < .05, n.s. *P* > .05).

There was an overall main effect of treatment on locomotor activity in mice following systemic administration of alcohol and intra‐VTA NMU pretreatment (*F*_[3,50]_ = 4.74, *P* = .0055). Post‐hoc analysis showed that compared with vehicle (n = 12), alcohol caused locomotor stimulation in both vehicle (*P* < .05, n = 14) and NMU (*P* < .05, n = 14) pretreated mice (Figure 4A). There was no difference in locomotor activity response in vehicle‐alcohol treated mice and NMU‐alcohol treated mice (*P* > .05), showing that intra‐VTA NMU administration does not attenuate the alcohol‐induced locomotor stimulation. This dose of NMU (n = 14) had no effect per se on locomotor activity compared with vehicle treatment (*P* > .05). Due to misplacement, four mice were excluded in the vehicle‐vehicle group, and two mice were excluded in the vehicle‐alcohol, NMU‐vehicle, NMU‐alcohol groups.

NMU administration into aVTA does not affect reward‐dependent memory retrieval of alcohol reward in the CPP test in mice (*P* = .9182; Figure 4B, n = 8 per group). Control experiments showed that intra‐VTA NMU (−2 ± 7%, n = 7) administration did not affect CPP per se compared with vehicle treatment (7 ± 11%, n = 7; *P* = .4646). Due to misplacement, one mouse was excluded in the vehicle‐vehicle and vehicle‐NMU groups.

There was no difference in peanut butter intake between NMU (n = 15) and vehicle (n = 14) treatment into the aVTA (*P* = .6327, Figure 4C). On the contrary, bilateral administration of NMU into aVTA reduced chow intake (*P* = .0387, Figure 4D). Due to misplacement, two mice were excluded in the vehicle group, and one mouse was excluded in the NMU group.

### 3.6 Outcomes of intra‐LDTg administration of NMU on alcohol‐induced locomotor stimulation, memory retrieval of alcohol reward in the CPP paradigm as well as food intake in mice

**Figure 5:**
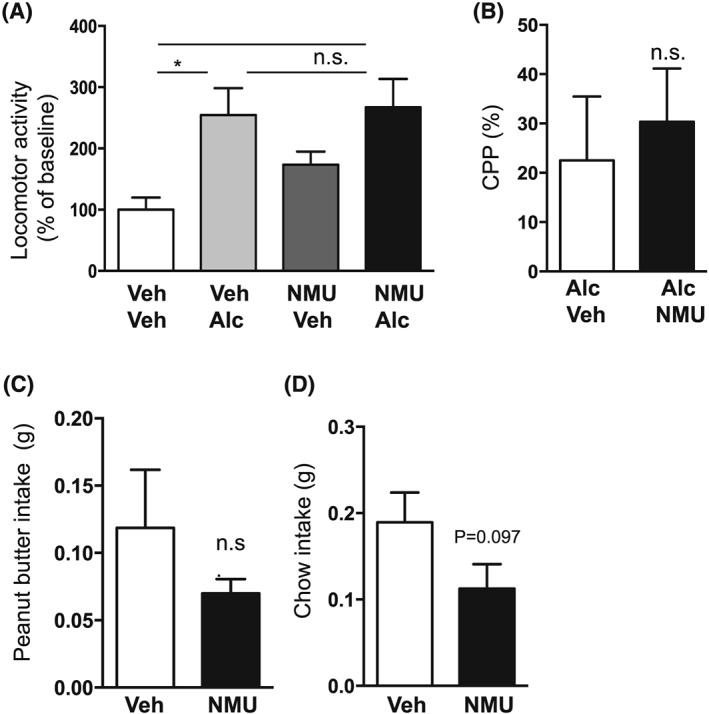
Outcomes of neuromedin U (NMU) infusion into the laterodorsal tegmental area (LDTg) on alcohol‐related behaviours and food intake in mice. A, Infusion of NMU (250 ng per side) into the LDTg did not affect alcohol (Alc)‐induced (1.75 g/kg, ip) locomotor stimulation compared with vehicle (Veh). B, Infusion of NMU into the LDTg did not alter the alcohol‐induced conditioned place preference (CPP) and C, had no effect on peanut butter intake in mice. D, There was a tendency in reduction in chow intake following NMU infusion into the LDTg. (Data are presented as mean ± SEM, *P < .05, **P < .01, n.s. *P* > .05).

An overall main effect of treatment on locomotor activity following systemic administration of alcohol and pretreatment of intra‐LDTg NMU (*F*_[3,57]_ = 4.79, *P* = .0048) was noted. As shown in Figure 5A, post‐hoc analysis revealed that in comparison with vehicle (n = 15), alcohol caused locomotor stimulation in vehicle (*P* < .05, n = 15) and NMU (*P* < .01, n = 16) pretreated mice, and this alcohol response was similar in both groups (*P* > .05), demonstrating that intra‐LDTg NMU administration does not attenuate alcohol‐induced locomotor stimulation. The selected dose of NMU (n = 15) had no effect per se on locomotor activity compared with vehicle treatment (*P* > .05). Due to misplacement, one mouse was excluded in the vehicle‐alcohol, NMU‐vehicle, and NMU‐alcohol groups.

There was no difference in alcohol response in the CPP paradigm in mice treated with NMU or vehicle into the LDTg (*P* = .6521; n = 6 in both groups, Figure 5B). Control experiments showed that there was no effect of intra‐LDTg NMU administration on CPP per se (−14 ± 6%, n = 8) compared with vehicle (6 ± 14%, n = 8; *P* = .6006). Due to misplacement, two mice were excluded in the alcohol‐vehicle and alcohol‐NMU groups.

Compared with vehicle, NMU into the LDTg had no effect on peanut butter intake (*P* = .2812; n = 15 per group, Figure 5C). There was a tendency to reduce chow intake (*P* = .0968, Figure 5D) following intra‐LDTg‐NMU. Due to misplacement, one mouse was excluded in each group.

## 4 DISCUSSION

The present study builds upon previous evidence showing a link between central NMU and reinforcement,^4^, ^5^, ^7^, ^21^, ^22^, ^25^, ^34^ by identifying brain region specific NMU signalling that modulates alcohol‐mediated behaviours. Striatum is an anatomically and functionally heterogeneous structure, where NAc shell is thought to contribute to the reinforcing properties and motivational aspects of alcohol.^35^, ^36^, ^37^, ^38^ The present findings reveal that infusion of NMU into the NAc shell prevents acute effects of alcohol as primarily measured by alcohol‐induced locomotor stimulation and formation of alcohol reward‐dependent memory in mice. A role of NMUR2‐NAc in the acute rewarding effects of addictive drugs is provided by data showing that NMU infusion into NAc shell attenuates amphetamine induced locomotor sensitisation^25^ and that the ability of acute cocaine to induce a locomotor stimulation is negatively associated with the expression of accumbal *NMUR2*.^39^ In addition, NMU into the NAc shell reduces alcohol intake in rats consuming alcohol for 12 weeks. However, there were no differences in *NMU* or *NMUR2* expression in NAc between high and low alcohol‐consuming rats. Collectively, this implicates that accumbal NMU signalling regulates the reinforcing properties of addictive drugs in rodents. Albeit the underlying mechanisms remain unknown, a tentative functional output is provided by the findings that activation of accumbal *NMUR2* expressed on GABAergic neurons projecting from dorsal raphe to NAc shell,^4^ selectively decreases GABA release in response to NMU.^4^ This GABA release may inhibit the indirect dopamine D2 pathway, which activates the mesolimbic dopamine system,^40^ consequently attenuating alcohol‐mediated behaviours. This possibility is substantiated by the findings that decreased GABA transmission in the NAc shell reduces alcohol consumption in rats.^41^, ^42^, ^43^ Possible support for the involvement of NAc shell as a mediator of the NMU‐alcohol link is provided by higher *cFos* expression^11^ in acute alcohol‐challenged rats that are pretreated with NMU compared with vehicle. Supportively, accumbal *cFos* expression is elevated following NMU administration into the third ventricle^44^ or NAc shell.^4^ A possible mechanism could lie in that accumbal NMU‐infusion decreases GABA release,^4^ which causes neuronal disinhibition, subsequently leading to increased neuronal activity. In addition, *cFos* expression might be influenced by intracellular pathways such as Gaq/11 and Gas as well as ERK1 and ERK2, which are activated by NMU (for review, see Gajjar and Patel^45^). Considerably, a different pattern of *cFos* activation might be obtained in mice or rats consuming alcohol for prolonged periods of time. The importance of the NMU‐NAc reinforcement link is further substantiated by previous findings that accumbal NMU infusion prevents amphetamine‐induced locomotor stimulation^25^ and cocaine‐induced sensitization^4^ in mice. In addition, our food choice studies show that NMU into the NAc shell, selectively reduces palatable and rewarding peanut butter consumption, further supporting a role of NMU‐NAc in reward processes. We here show that intra‐NAc NMU attenuates acute and chronic effects of alcohol; however, the origin of NMU detected in the NAc^9^ could include neuronal production or peripheral release. As the circulating NMU levels are low due to rapid degradation,^46^ the possibility that NMU is produced locally in distinct brain regions appears more likely. NMU coreleasing afferents of the lateral hypothalamus,^5^ an area that regulates drug reinforcement through its NAc projection,^47^, ^48^ may provide one tentative origin.

A differential NMU signalling in dorsal striatum of high compared with low alcohol‐consuming rats was revealed, as reflected by elevated *NMU* and reduced *NMUR2* expression. The physiological outcome of these correlational studies is limited, as no baseline gene expression is provided and protein levels are not studied. However, this NMU/NMUR2 imbalance, possibly causes high alcohol consumption as a result of reduced activity of the mesolimbic dopamine system^40^ because of chronic reduction in striatal GABA levels.^4^ Studies suggest that habit formation of compulsive alcohol seeking following extended exposure to alcohol involves dorsal striatum,^49^, ^50^, ^51^ implying that this local NMU signalling may contribute to the manifestation of AUD. Added context for this dorsal striatum correlation is provided by the findings that cocaine sensitisation eliminates the negative association between accumbal *NMUR2* expression and the locomotor stimulatory effect of acute cocaine.^39^ Interestingly, enhanced activity in the dorsal striatum depends on changes in the dopamine activity in NAc,^52^ raising the possibility that an interconnection of NMU signalling within both areas regulates alcohol‐mediated behaviours. As the present study revealed a correlational association between dorsal striatum and alcohol intake, it should be considered as an initial indication for the importance of NMU signalling within the dorsal striatum for alcohol‐mediated behaviour. Therefore, upcoming studies should investigate the role of the medial and lateral dorsal striatum as well as the connection to NAc shell, for this NMU‐alcohol link in detail.

Both the aVTA as well as LDTg have established roles in alcohol reinforcement.^14^, ^15^, ^16^ However, NMU administration into either of the aforementioned areas did not modulate the acute effects of alcohol in mice, as measured by alcohol‐induced locomotor stimulation and reward‐dependent memory retrieval in the CPP model. The findings that NMU infusion into either area did not alter peanut butter intake in mice, further support that NMU signalling within the aVTA and LDTg does not modulate reward. Nevertheless, NMU into these areas possibly modulates alcohol intake in rats. It is also plausible that the behavioural responses to NMU into the posterior part of the VTA, where rats self‐administer alcohol,^53^, ^54^ may be diverge. Another physiological role of NMU signalling within the aVTA is however most likely as we here show that the aVTA expresses *NMU* as well as *NMUR2* and that NMUR2 was recently detected in the VTA.^10^ Indeed, both aVTA and LDTg were identified as novel areas, where NMU acts to reduce 4‐hour chow intake in mice, further confirming its anorexigenic properties.^2^, ^3^, ^22^ Support for a modulatory role of NMU signalling in the VTA for feeding is provided by the recent study demonstrating a negative correlation between synaptosomal NMUR2 protein in the VTA and binge intake of a high fat mixture.^10^ As the expression of *NMU* or *NMUR2* in the LDTg has not been studied to date and the physiological relevance of the present data should be investigated further, in addition, the exact mechanisms underlying these behavioural responses to NMU in the LDTg are not clear and the possibility should be considered that NMU might have unselective receptor effects in this area. However, this appears less likely since reversed pharmacology studies have established NMU as the ligand for NMUR2.^2^ The feeding suppressive effect on chow in this study, does not appear to involve NAc shell, as NMU into this area did not influence chow intake in mice or in rats consuming alcohol for 12 weeks. On the other hand, protein expression of NMUR2 is positively correlated with binge‐type eating of high fat food in rats.^10^ However, it is possible that NMU does not reduce food intake in rodents exposed to a choice between chow and a rewarding stimulus, such as peanut butter or alcohol. Therefore, the role of accumbal NMUR2 in additional feeding studies including alcohol, chow, and peanut butter would be an interesting future directive.

The present study provides compelling support for the role of NAc shell as a novel NMU site of action; however, certain limitations possibly influencing the obtained data should be taken into consideration. While intracranial infusions may induce tissue damage, the inclusion of vehicle controls in this study diminishes this possibility. Potential drug diffusion outside of the target brain areas may have an effect; however, in animals with misplaced guides, as opposed to guides targeting the area, no effect of the drug was observed. Effects on gross behaviour could influence the obtained results in mice as well as in rats. In mice, this appears less likely since we here show that NMU infusion into the selected brain regions does not influence locomotor activity or CPP per se. Albeit no visual effect on gross behaviour was observed in alcohol consuming or alcohol naïve rats in addition to NAc‐NMU does not influencing locomotor activity in rats,^4^ NMU possibly alters locomotor activity in rats consuming alcohol in the intermittent exposure paradigm. Another possible limitation could be that both mice and rats were used. However, acute behavioural effects of alcohol in mice and alcohol intake in rats are reduced by central NMU.^7^ Despite NAc shell being identified as an important area for alcohol‐mediated behaviours, NMUR2 in other brain regions not investigated here might be involved. Another tentative limitation could be that food intake in vehicle‐treated controls is not stable across the feeding studies. As mice most likely are influenced differently by local injections in the NAc shell, aVTA, and LDTg, response to NMU is always compared with the corresponding vehicle. Moreover, the mice food intake results could possibly be influenced by prior food exposure to chow or peanut butter. A limitation with the *cFos* study is the absence of vehicle‐vehicle and NMU‐vehicle control groups, and therefore, this design only allows conclusions regarding the NMU‐alcohol interaction rather than their independent effects.

In agreement with previous data,^7^, ^25^ the present study highlights the emerging role of NMU signalling in reward and alcohol consumption. Additionally, it expands on the present knowledge by showing that the striatal NMU neurocircuitry is closely linked to alcohol‐mediated behaviours and peanut butter intake, a palatable and rewarding food, in rodents. Furthermore, NMU into aVTA or LDTg reduced chow consumption but did not modulate alcohol‐related behaviours. Collectively, the above suggest that NMU signalling within different brain areas modulates the differential roles of this neuropeptide.

## CONFLICT OF INTEREST

The remaining authors declare no conflict of interest.

## AUTHORS CONTRIBUTIONS

Britt‐Mari Larsson and Minja Mandrapa are gratefully acknowledged for valuable technical assistance. The study is supported by grants from the Swedish Research Council (2015‐03219), Swedish Society for Medical Research, The Swedish brain foundation, LUA/ALF (grant no. 148251) from the Sahlgrenska University Hospital. E.J. has received financial support from the Novo Nordisk Foundation for another project. This does not alter the authors' adherence to any of the journal's policies on sharing data and materials.

## Supporting information

Data S1.
Supporting information

Data S2.
Supporting information

Data S3.
Supporting information

Data S4.
Supporting information
