# Supplementary material for: Brain region‐specific neuromedin U signalling regulates alcohol‐related behaviours and food intake in rodents
Source: Addict Biol. 2019 May 8;25(3):e12764. doi: 10.1111/adb.12764 (PMC7187236; doi:10.1111/adb.12764)
Supplement: Supplementary file 1 — Data S1. Supporting information [file ADB-25-e12764-s001.pptx]

## Slide 1
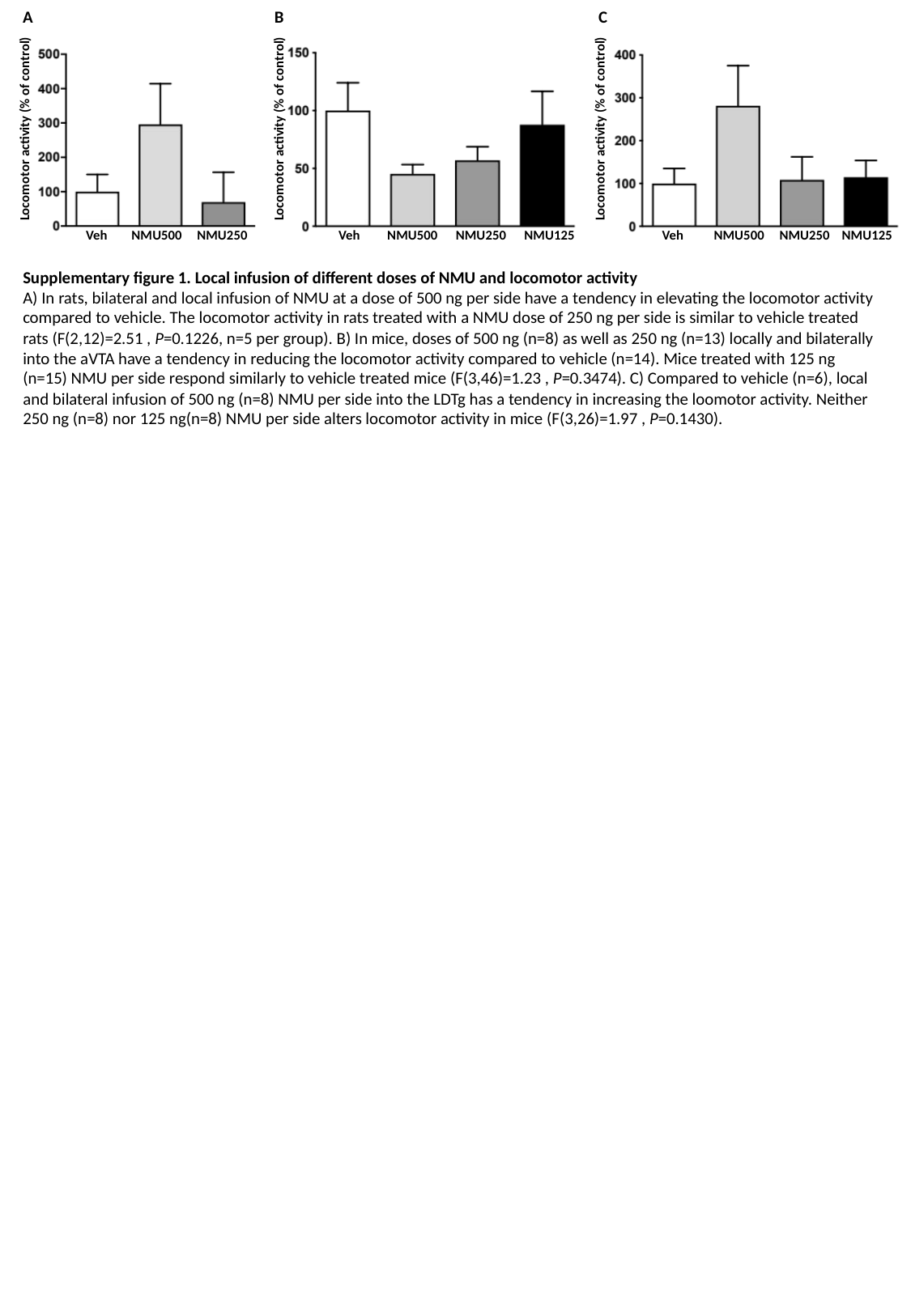

A B C
Locomotor activity (% of control)
Locomotor activity (% of control)
Locomotor activity (% of control)
Veh NMU500 NMU250
Veh NMU500 NMU250 NMU125
Veh NMU500 NMU250 NMU125
Supplementary figure 1. Local infusion of different doses of NMU and locomotor activity
A) In rats, bilateral and local infusion of NMU at a dose of 500 ng per side have a tendency in elevating the locomotor activity compared to vehicle. The locomotor activity in rats treated with a NMU dose of 250 ng per side is similar to vehicle treated rats (F(2,12)=2.51 , P=0.1226, n=5 per group). B) In mice, doses of 500 ng (n=8) as well as 250 ng (n=13) locally and bilaterally into the aVTA have a tendency in reducing the locomotor activity compared to vehicle (n=14). Mice treated with 125 ng (n=15) NMU per side respond similarly to vehicle treated mice (F(3,46)=1.23 , P=0.3474). C) Compared to vehicle (n=6), local and bilateral infusion of 500 ng (n=8) NMU per side into the LDTg has a tendency in increasing the loomotor activity. Neither 250 ng (n=8) nor 125 ng(n=8) NMU per side alters locomotor activity in mice (F(3,26)=1.97 , P=0.1430).

## Slide 2
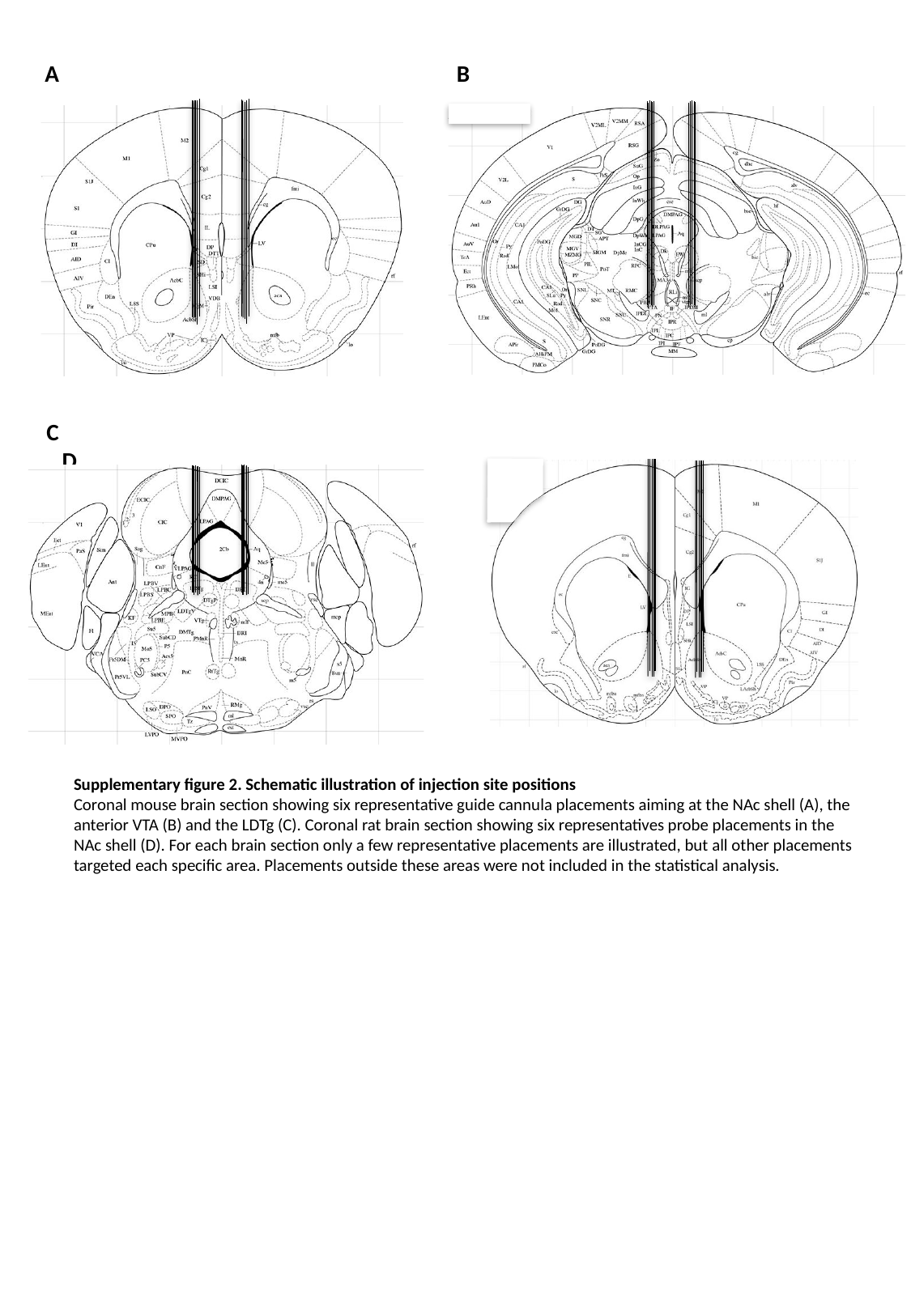

A 	 B
C 			 D
Supplementary figure 2. Schematic illustration of injection site positions
Coronal mouse brain section showing six representative guide cannula placements aiming at the NAc shell (A), the anterior VTA (B) and the LDTg (C). Coronal rat brain section showing six representatives probe placements in the NAc shell (D). For each brain section only a few representative placements are illustrated, but all other placements targeted each specific area. Placements outside these areas were not included in the statistical analysis.

## Slide 3
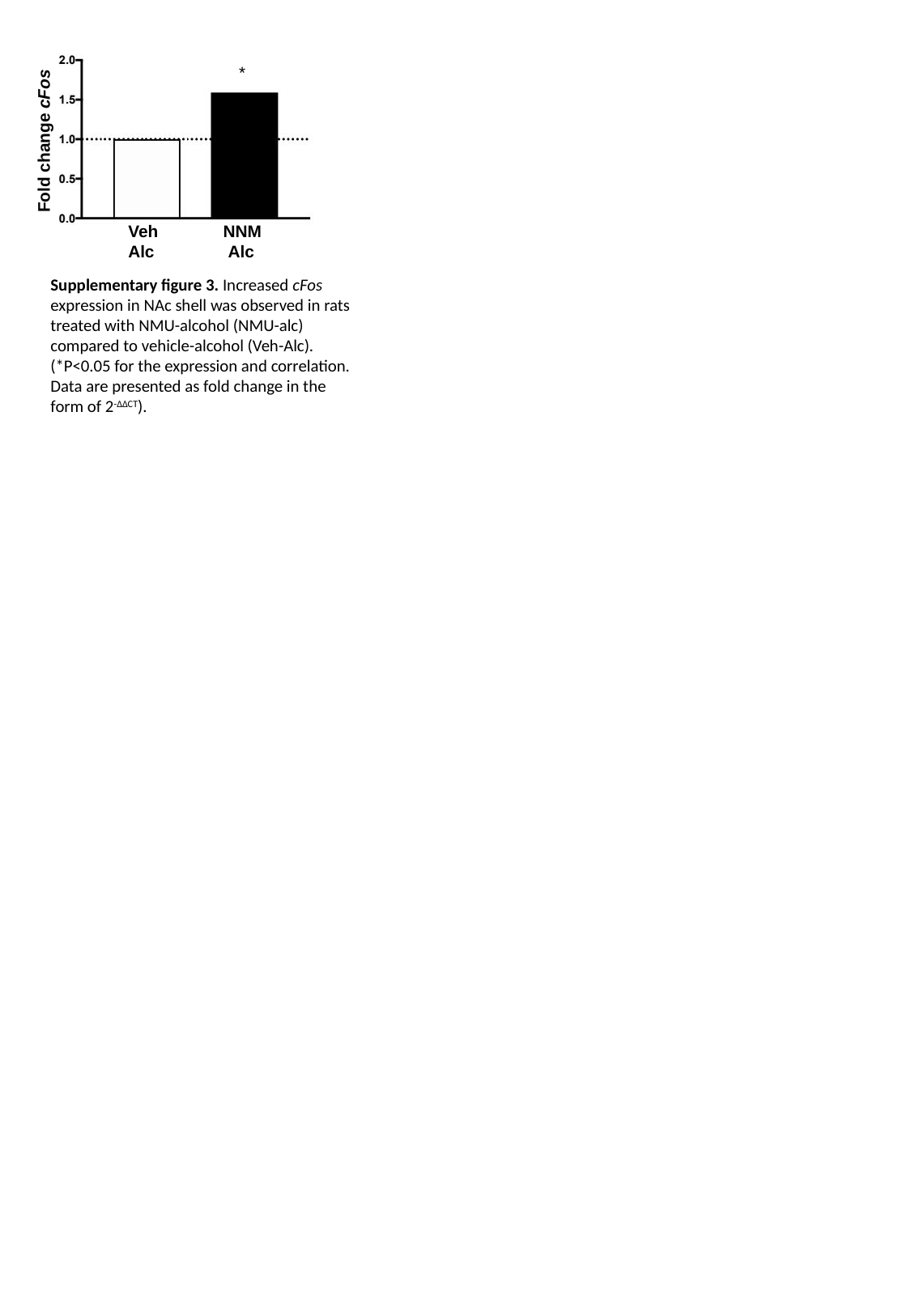

*
Fold change cFos
Veh NNM Alc Alc
Supplementary figure 3. Increased cFos expression in NAc shell was observed in rats treated with NMU-alcohol (NMU-alc) compared to vehicle-alcohol (Veh-Alc). (*P<0.05 for the expression and correlation. Data are presented as fold change in the form of 2-ΔΔCT).
