# Supplementary material for: Brain region‐specific neuromedin U signalling regulates alcohol‐related behaviours and food intake in rodents
Source: Addict Biol. 2019 May 8;25(3):e12764. doi: 10.1111/adb.12764 (PMC7187236; doi:10.1111/adb.12764)
Supplement: Supplementary file 2 — Data S2. Supporting information [file ADB-25-e12764-s002.docx]

**Supplementary material 1**

***Guide implantation***

The rodent was anesthetized with isofluran (Isofluran Baxter, Apoteket AB, Gothenburg, Sweden) using a pump (Univentor 400 Anaesthesia Unit, Univentor Ldt., Zejtun, Malta), placed in a stereotaxic frame (David Kopf Instruments; Tujunga, CA, USA) and kept on a heating pad to prevent hypothermia. Xylocain (10 mg/ml) adrenalin (5 μg/ml) (Pfizer Inc, Apoteket AB, Gothenburg, Sweden) applied locally on the skull surface, was used as local anesthetics and carprofen (Rimadyl**®**, 5 mg/kg ip, Astra Zeneca; Gothenburg, Sweden) was used to relieve pain. The skull bone was exposed and two holes for the bilateral guide (stainless steel, length 10 mm, with an o.d./i.d. of 0.6/0.45 mm), allowing local injections, and one hole for the anchoring screw were drilled and the guide were anchored to the screw and the skull bone with dental cement (DENTALON^®^ plus; Agntho’s AB, Lidingö, Sweden). Coordinates used for NAc shell, anterior VTA and LDTg were based on previous studies in mice and rats (Jerlhag et al., 2012; Prieto-Garcia et al., 2015). Coordinates (anterior, lateral to midline, below brain surface) used in mice: NAc shell +1.4 mm, ±0.6 mm, - 1.0 mm; anterior/medial VTA -3.4 mm, ±0.5 mm, -1.0 mm; LDTg -5.0 mm, ±0.5 mm, -1.0 mm (Franklin and Paxinos, 1996). The mice were housed individually following surgery. At the time of the experiment, a cannula was extended another 3.7 mm, 3.8 mm or 2.2 mm ventrally beyond the tip of the guide, aiming at the NAc, VTA and LDTg respectively. In rats, the following coordinates for NAc shell were used: 1.85 mm anterior to the bregma, ±1.0 mm lateral to the midline and -1.0 mm below the surface of the brain (Paxinos and Watson, 1998). A cannula extended another 6.8 mm beyond the tip of the guide aiming at NAc was used at the time of the experiment. The injection sites were verified following the termination of the experiment as described previously (Jerlhag et al., 2012; Prieto-Garcia et al., 2015) and only animals with correct placements were included in the statistical analysis. In all experiments one hour before the injections, a dummy cannula was inserted through the guide to remove clotted blood and damper spreading depression. Mice and rats with placements outside of the area of interest were excluded from the analysis. Albeit the possibility that local infusions might spread to surrounding areas, back flow into the cannula or diffusion into ventricles this appears less likely since we throughout the years have established that misplaced injections of different pharmacological agents does not attenuate alcohol- or drug-related behaviours.

**References**

Franklin KBJ, Paxinos G (1996) *The Mouse Brain in Stereotaxic Coordinates*. Academic Press: New York.

Jerlhag E, Janson AC, Waters S, Engel JA (2012) Concomitant release of ventral tegmental acetylcholine and accumbal dopamine by ghrelin in rats. PLoS One 7:e49557.

Paxinos G, Watson C (1998) *The brain stereotaxic coordinates*. Academic Press: New York.

Prieto-Garcia L, Egecioglu E, Studer E, Westberg L, Jerlhag E (2015) Ghrelin and GHS-R1A signaling within the ventral and laterodorsal tegmental area regulate sexual behavior in sexually naive male mice. Psychoneuroendocrinology 62:392-402.
