# Supplementary material for: Brain region‐specific neuromedin U signalling regulates alcohol‐related behaviours and food intake in rodents
Source: Addict Biol. 2019 May 8;25(3):e12764. doi: 10.1111/adb.12764 (PMC7187236; doi:10.1111/adb.12764)
Supplement: Supplementary file 3 — Data S3. Supporting information [file ADB-25-e12764-s003.docx]

**Supplementary material 2**

All surfaces and instruments were cleaned with RNase decontamination solution (RNase *Zap***™** , Invitrogen**™**, ThermoFisher Scientific, Wilmington, DE, USA). For RNA tissue preparation, the brain samples were homogenized by adding a TissueLyser II (Qiagen, Hilden, Germany). The total RNA was extracted using the RNeasy Lipid Tissue Mini kit (Qiagen) and samples were loaded on the QIAcube^TM^ (Qiagen, Hilden, Germany) for automated RNA extraction, following the manufacturer’s protocols. The quality and concentration of RNA were assessed in 1μl of the final RNA solution, using a NanoDrop 1000 Spectrophotometer (ThermoFisher Scientific, Wilmington, DE, USA). RNA concentration was calculated equally for all samples at 1000 ng per sample. After dilution with Milli-Q^TM^ (Millipore Corporation, Billerica, MA, USA) water, the samples were loaded in duplicates in a 96 well plate (Sarstedt AG & Co, Nümbrecht, Germany) and were prepared for a 20 μl reverse transcription reaction into cDNA using the QuantiTect Reverse Transcription kit (Qiagen, Hilden, Germany) as per manufacturer’s instructions. The quantitative real-time PCR (qRT-PCR) analysis was performed in the facilities of TATAA Biocenter AB Gothenburg, Sweden. Briefly, the samples were corrected for gDNA contamination using the ValidPrime^TM^ (TATAA Biocenter AB) technology and gene expression analysis was performed using the qRT-PCR instrument IntelliQube^TM^ (Douglas Scientific, Alexandria, MN, USA).
