# Supplementary material for: Brain region‐specific neuromedin U signalling regulates alcohol‐related behaviours and food intake in rodents
Source: Addict Biol. 2019 May 8;25(3):e12764. doi: 10.1111/adb.12764 (PMC7187236; doi:10.1111/adb.12764)
Supplement: Supplementary file 4 — Data S4. Supporting information [file ADB-25-e12764-s004.docx]

**Supplementary table 1. *NMU* expression in reward-related brain areas of low and high alcohol-consuming rats after twelve weeks of voluntary alcohol consumption (cut of 3.5 g/kg).**

| Brain region | Low alcohol- consuming rats | High alcohol- consuming rats | t | df | *P*-value^1^ |
| --- | --- | --- | --- | --- | --- |
| Nucleus accumbens | 0.91±0.06; n=21 | 1.00± 0.01; n=19 | 1.132 | 38 | 0.265 |
| Ventral tegmental area | 1.10±0.10; n=25 | 1.05±0.08; n=21 | 0.387 | 44 | 0.701 |
| Prefrontal cortex | 1.08±0.08; n=28 | 0.97±0.07; n=20 | 0.089 | 46 | 0.380 |
| Amygdala | 0.97±0.03; n=26 | 1.02±0.07; n=19 | 0.725 | 43 | 0.472 |
| Hippocampus | 1.07±0.83; n=23 | 1.37±0.15; n=20 | 1.969 | 41 | 0.056 |

Data presented as ΔC_T_ values ± SEM. ^1^unpaired t-test.

**Supplementary table 2. *NMUR2* expression in reward-related areas of low and high alcohol-consuming rats after twelve weeks of voluntary alcohol consumption (cut of 3.5 g/kg).**

| Brain  region | Low alcohol- consuming rats | High alcohol- consuming rats | t | df | *P*-value^1^ |
| --- | --- | --- | --- | --- | --- |
| Nucleus accumbens | 1.17±0.15; n=17 | 0.96±0.13; n=13 | 0.986 | 28 | 0.333 |
| Ventral tegmental area | 1.03±1.36; n=16 | 1.07±0.14; n=15 | 0.227 | 29 | 0.822 |
| Prefrontal cortex | 1.32± 0.20; n=24 | 1.42±0.23; n=20 | 0.338 | 42 | 0.737 |
| Amygdala | 1.04± 0.11; n=19 | 1.14±0.21; n=15 | 0.458 | 32 | 0.650 |
| Hippocampus | 1.38± 0.20; n=20 | 1.15±0.29; n=19 | 0.808 | 37 | 0.424 |
| Dorsal striatum | 1.08± 0.85; n=26 | 0.89±0.14; n=22 | 1.197 | 46 | 0.237 |

Data presented as ΔC_T_ values ± SEM. ^1^unpaired t-test.
